# Repeatability of Pulse Oximetry Measurements in Children During Triage in 2 Ugandan Hospitals

**DOI:** 10.9745/GHSP-D-22-00544

**Published:** 2023-08-28

**Authors:** Ahmad Asdo, Alishah Mawji, Collins Agaba, Clare Komugisha, Stefanie K. Novakowski, Yashodani Pillay, Stephen Kamau, Matthew O. Wiens, Samuel Akech, Abner Tagoola, Niranjan Kissoon, J. Mark Ansermino, Dustin Dunsmuir

**Affiliations:** a Department of Anesthesiology, Pharmacology & Therapeutics, University of British Columbia, Vancouver, Canada.; b Institute for Global Health at BC Children’s and Women’s Hospital, Vancouver, Canada.; c World Alliance for Lung and Intensive Care Medicine in Uganda, Kampala, Uganda.; d Kenya Medical Research Institute-Wellcome Trust Research Programme, Nairobi, Kenya.; e Department of Pediatrics, Jinja Regional Referral Hospital, Jinja, Uganda.; f Department of Pediatrics, University of British Columbia, Vancouver, Canada.

## Abstract

**Background:**

In low- and middle-income countries, health workers use pulse oximeters for intermittent spot measurements of oxygen saturation (SpO2). However, the accuracy and reliability of pulse oximeters for spot measurements have not been determined. We evaluated the repeatability of spot measurements and the ideal observation time to guide recommendations during spot check measurements.

**Methods:**

Two 1-minute measurements were taken for the 3,903 subjects enrolled in the study conducted April 2020–January 2022 in Uganda, collecting 1 Hz SpO2 and signal quality index (SQI) data. The repeatability between the 2 measurements was assessed using an intraclass correlation coefficient (ICC), calculated using a median of all seconds of non-zero SpO2 values for each recording (any quality, Q1) and again with a quality filter only using seconds with SQI 90% or higher (good quality, Q2). The ICC was also recalculated for both conditions of Q1 and Q2 using the initial 5 seconds, then the initial 10 seconds, and continuing with 5-second increments up to the full 60 seconds. Lastly, the whole minute ICC was calculated with good quality (Q2), including only records where both measurements had a mean SQI of more than 70% (Q3).

**Results:**

The repeatability ICC with condition Q1 was 0.591 (95% confidence interval [CI]=0.570, 0.611). Using only the first 5 seconds of each measurement reduced the repeatability to 0.200 (95% CI=0.169, 0.230). Filtering with Q2, the whole-minute ICC was 0.855 (95% CI=0.847, 0.864). The ICC did not improve beyond the first 35 seconds. For Q3, the repeatability rose to 0.908 (95% CI=0.901, 0.914).

**Conclusions:**

Training guidelines must emphasize the importance of signal quality and duration of measurement, targeting a minimum of 35 seconds of adequate-quality, stable data. In addition, the design of new devices should incorporate user prompts and force quality checks to encourage more accurate pulse oximetry measurements.

## Abstract (precis)

This analysis of spot measurements of oxygen saturation in children during routine triage in Ugandan hospitals indicates the importance of signal quality and duration of measurement in achieving repeatable pulse oximetry measurements.

## Teaser

**Key Findings:**

- Repeatability of oxygen saturation level measurements is critical for making optimal clinical decisions but also essential when performing device validation.
- In this assessment at 2 hospitals in Uganda, shorter periods of observation, lower signal quality, and lower oxygen saturation levels reduced repeatability of pulse oximetry measurements.
- Repeatability increased as the duration of recordings increased in length but plateaued at 35 seconds.

**Key Implications:**

- Health workers who perform pulse oximetry should average oxygen saturation readings over at least 35 seconds to obtain a repeatable measurement.
- To meet the requirement necessary to obtain adequate quality data, pulse oximeters must display signal adequacy. Device manufacturers can enforce this practice with improved device design.

## INTRODUCTION

Pulse oximetry is a noninvasive light-through-tissue technology that uses red and infrared light to measure oxygen saturation (SpO2). It is commonly used in emergency and intensive care departments, during surgery, and at home to monitor the oxygenation status of patients. The World Health Organization’s updated pediatric Emergency Triage, Assessment, and Treatment guidelines recommend using pulse oximetry to determine the presence of hypoxemia in all children with emergency signs listed in the guidelines. They also recommend oxygen supplementation if SpO2 levels are below 90% or 94% (depending on the presence of respiratory distress and other clinical signs).^1^ When coupled with a reliable oxygen supply, monitoring SpO2 with pulse oximetry in low- and middle-income countries (LMICs) has been shown to reduce mortality from pneumonia by as much as 35%.^2^ When combined with clinical signs, a low oxygen saturation level is a strong predictor of the need for hospital admission^3^^,^^4^ or death^5^ for children with infectious illnesses in LMICs.

The durability and replacement costs of pulse oximeter sensors have been a limiting factor in the sustainable implementation of pulse oximetry as a clinical tool in LMICs.^2^ The increasing recognition of the importance of supplemental oxygen therapy and the finding that the current devices often do not perform well when used by health care workers has catalyzed the development of new pulse oximeters better suited to low-resource settings.^6^^,^^7^ Due to limited device availability, a lack of trained personnel, and excessive patient load, spot monitoring is likely to be more common than continuous SpO2 monitoring for children in resource-limited environments.^8^

Due to limited pulse oximeter availability, lack of trained personnel, and excessive patient load, spot monitoring is likely to be more common than continuous SpO2 monitoring for children in resource-limited environments.

The accuracy of the Masimo iSpO2 module technology used is ±2% under no motion conditions and low perfusion conditions and ±3% under motion conditions.^9^ This is similar to the Lifebox pulse oximeter and meets the current International Organization for Standardization standards of less than 3%.^10^^–^^12^ The reliability of spot measurements is uncertain, though. One approach is to take multiple SpO2 readings to inform clinical decisions of high importance.^13^ However, there has been a lack of research on how to achieve the most reliable SpO2 value within the shortest time and with the lowest training overhead for health care workers.

We aimed to assess the repeatability of SpO2 spot measurements in routine triage of children during a clinical study in a low-resource environment, where repeatability is defined as the likelihood of getting the same results of a measurement with the same operator, same device, and same patient within a short period of time.^14^

## METHODS

### Study Cohort

This study is a planned secondary analysis of pulse oximetry data from an interrupted time series study to validate a digital triaging platform called Smart Triage (ClinicalTrials.gov Identifier: NCT04304235).^15^^,^^16^ We obtained repeated pulse oximetry measurements from 3,903 children presenting to the outpatient departments of Jinja Regional Referral Hospital (2,141 patients) and Gulu Regional Referral Hospital (1,762 patients) in Uganda. The data collection sites were at an altitude of 1100–1200 m above sea level. All children seeking medical treatment for an acute systemic illness during the baseline phase of Smart Triage were eligible (April 2020 to March 2021 at Jinja Regional Referral Hospital and March 2021 to January 2022 at Gulu Regional Referral Hospital). Children aged from birth to 19 years were recruited based on the hospitals’ age threshold for pediatric admissions. Children seeking care for elective procedures or clinical review appointments were excluded. Participation was voluntary, and a parent or guardian provided written informed consent before enrollment. Assent was required from children aged older than 8 years.

### Data Collection

A study nurse collected more than 200 variables, including pulse oximetry and other clinical signs, symptoms, and sociodemographic variables.^17^ For each child, 2 1-minute pulse oximetry spot measurements were collected using a customized mobile application and the Masimo iSpO2 Pulse Oximeters (2020, Masimo Corporation, United States) with age-appropriate M-LNCS DCIP (pediatric) and M-LNCS YI (multisite, neonate) sensors and micro-USB connected directly to an Android data collection tablet. The application was designed to encourage the user to obtain the highest quality recording using background color coding and forcing functions that minimized recordings of low-quality data. The user connected the probe and waited for the color-coded quality signal, which appeared as a green background behind the waveform, before beginning the recording. The user initiated the recording when an adequate signal quality was obtained. Trend values, including heart rate (HR), SpO2, and signal quality index (SQI), were recorded at 1 Hz, and the raw plethysmograph waveform was recorded at 62.5 Hz.^18^ The oxygen saturation, heart rate, perfusion index, and plethysmograph waveform provided by the hardware and custom color-based graphical display of signal quality were displayed in the custom app. The SQI was calculated as a percentage using Masimo status flags (excess light, artifacts, low perfusion, pulse search, low signal identification, and quality indicator), the perfusion index, and the variability of SpO2 and HR trends (Supplement). The SQI for a spot measurement was calculated as the mean SQI from all the data. The time at the beginning of each measurement was recorded, which enabled the calculation of the time difference between the 2 measurements, given that each measurement lasted for 1 minute. The median SpO2 and HR values provided by the app for each 1-minute measurement were calculated using data with SQI≥90% (considered good quality). If there was no continuous 30-second period of high-quality data, the application prompted the user to perform an additional measurement. Any number of recordings could be done, but staff were trained to acquire 2 good recordings. Only the 2 highest-quality measurements were saved and then analyzed in this study.

### Data Analysis

Records containing at least 2 pulse oximetry measurements recorded less than 1 hour apart and with more than 80% of their variables present were analyzed. The measurement with the highest-quality SQI (SpO2-1) was compared to the measurement with the second highest quality SQI (SpO2-2). SpO2 values of 0 were ignored as these were seconds where the pulse oximeter failed to provide a value. The SQIs from the plethysmograph were summarized using histograms, and the median time difference between the 2 recordings was calculated.

The SpO2 for the spot measurements was recalculated from the 1 Hz data in 3 different ways, referred to as Q1-Q3. First, SpO2 dataset Q1 was calculated from the 1 Hz data using all records and all non-zero seconds of SpO2 data. This calculation is most reflective of real-world clinical applications in which devices provide SpO2 values whenever they can be calculated and health care workers use numbers directly from the devices without any intermediate averaging. The second SpO2 dataset (Q2) included all records and followed the data collection application rules of only using data with good quality (SQI≥90%). This calculation indicates the repeatability under optimal SQI conditions while not excluding any patients. The third SpO2 dataset (Q3) applied the good quality data rule from Q2 but only included records that had a mean SQI value (from all the data) of above 70% for both SpO2-1 and SpO2-2.

Agreement between the 2 SpO2 measurements using dataset Q1 was visualized using Bland Altman plots (SpO2 difference vs. mean of the 2 measurements) to identify any systematic error within the measurements and possible outliers. Outliers were visually identified from the plots and were defined as measurements with an SpO2 absolute difference of greater than 20%. The repeatability bias (Table 1)^19^ and the limits of agreement were calculated for datasets Q1 and Q2.

**TABLE 1.** Definitions^a^ Used in the Analysis of Pulse Oximetry Measurements for Children in Triage at 2 Ugandan Hospitals

**Term**	**Definition**
Repeatability	Precision under repeatability conditions. In this study, this is the repeatability of pulse oximetry (SpO2) on children.
Repeatability conditions	Conditions where independent test results are obtained with the same method on identical test items in the same laboratory by the same operator using the same equipment within short intervals of time.
Repeatability bias	The bias under repeatability conditions, considering 1 set of samples as the test and the other as the reference. In this study, for the purposes of bias, the highest-quality SpO_2_ recordings (SpO2-1) are considered the reference value and the second highest-quality recordings (SpO2-2) are considered the test results.
Accuracy	The closeness of agreement between a test result and the accepted reference value.
Bias	The difference between the expectation of the test results and an accepted reference value.

^a^ Derived from the International Organization of Standardization.^19^

Repeatability (Table 1) was assessed using the intraclass correlation coefficient (ICC) between the median SpO2 from SpO2-1 and SpO2-2 in a 2-way random effects model with a single rater. The ICC was calculated for all 3 datasets, and the Q1 and Q2 calculations were repeated for different spot measurement durations starting with the initial 5 seconds, then initial 10 seconds, and continuing with 5-second increases to the duration until the calculation included the full minute. We then plotted these 2 conditions as overlapping bar graphs to see the effect of signal quality and measurement duration on the repeatability. Data were analyzed using R software version 4.1.2.

### Ethical Approval

This study was approved by the institutional review boards at the University of British Columbia in Canada (ID: H19-02398; H20-00484), the Makerere University School of Public Health in Uganda, and the Uganda National Council for Science and Technology. The clinical trial was registered on March 11, 2020 (Clinical Trials.gov Identifier: NCT04304235).

## RESULTS

Of the 3,903 patients enrolled in the study, we excluded 80 patients due to missing paired measurements (n=58) or time difference between the measurements of longer than 1 hour (n=22), leaving 3,823 patients who were included in this analysis. The median age was 15 (interquartile range [IQR]: 26.6) months and 52% were male (Table 2).

**TABLE 2.** Demographic Variables of Patients Included in the Analysis of Pulse Oximetry Measurements for Children in Triage at 2 Ugandan Hospitals, April 2020–January 2022

	**Females**	**Males**	**Overall**
No. (%)	1,846 (48)	1,977 (52)	3,823 (100)
Median age, months	16.0	14.2	14.8
Interquartile range of age, months	28.4	24.6	26.6

**FIGURE 1:**
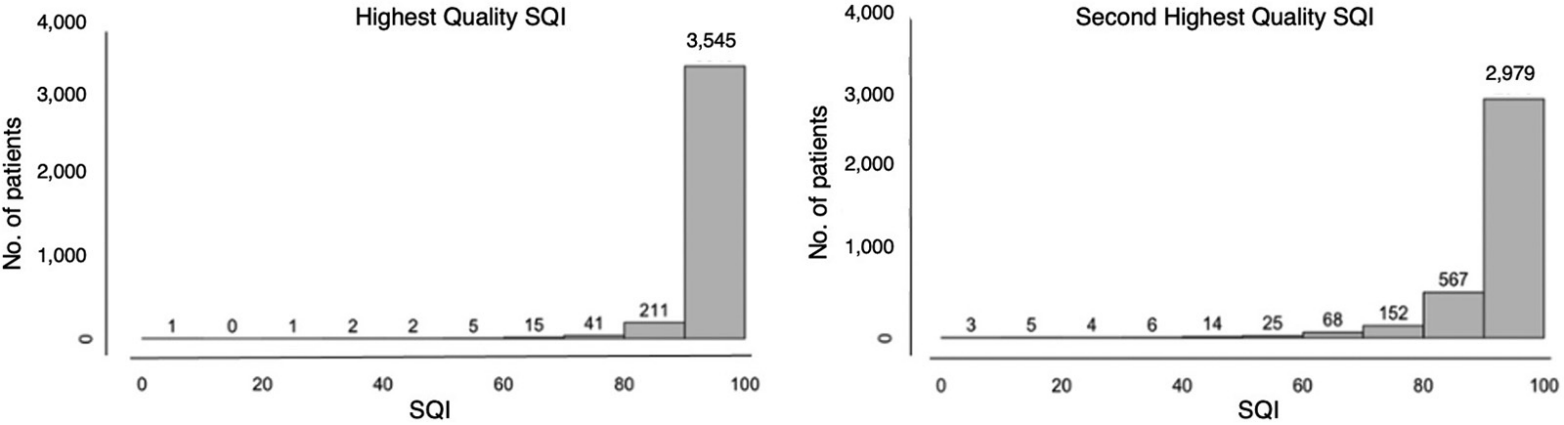
Histogram of Oxygen Saturation Readings Distributed for Highest- and Second Highest-Quality Oxygen Saturation Measurements Abbreviation: SQI, signal quality index.

The median SQI for all the data was 93% (IQR: 92%–97%) (Figure 1). Most (93%) of the highest quality observations (SpO2-1) from the 2-paired spot measurements had SQI≥90%. For the lower quality observation (SpO2-2), 78% of measurements had SQI≥90%. There was a median of 0.21 (IQR: 0.13–0.65) minutes between measurements and 95% of the measurements occurred less than 8 minutes apart.

In dataset Q1 (no quality consideration), both SpO2-1 and SpO2-2 had a median SpO2≥90% for 99.0% of the records. Two patients were identified as outliers, and both these patients had hypoxemia (SpO2<90%) for 1 of the 2 measurements.

When calculating the mean perfusion index per patient and averaging those indices across the entire population, the mean perfusion index for SpO2-1 was 3.2, whereas for SpO2-2, it was 2.9. The mean weight for the population was 11.3 kg, but this varied by age.

**FIGURE 2:**
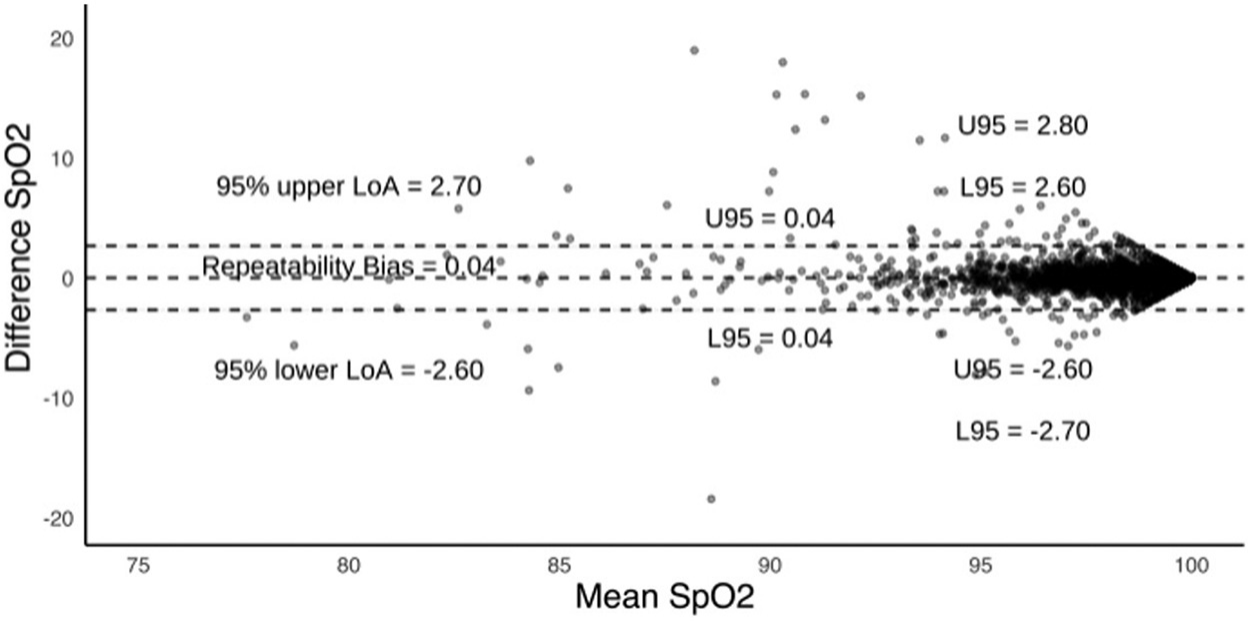
Bland Altman Plot for Oxygen Saturation Values^a^ Abbreviations: L95, lower limit of confidence interval; LoA, limit of agreement; U95, upper limit of confidence interval. ^a^N=3,823. Each data point is for a pair of recordings with the y-axis as difference (SpO2-1–SpO2-2) and the x-axis as the mean SpO2. Two outliers were identified and excluded from Figure 2 but included in further analysis because they met the inclusion criteria. Includes only SpO2>0.

The most extreme disagreements between SpO2-1 and SpO2-2 were observed when the mean of the 2 measurements was low (Figure 2). The repeatability bias of SpO2 was 0.04%. The spread between the upper and lower limits of agreement was 5.8%. Using Q2, the repeatability bias was 0.03%, and the spread of the upper and lower limits of agreement was 5.2%.

**FIGURE 3:**
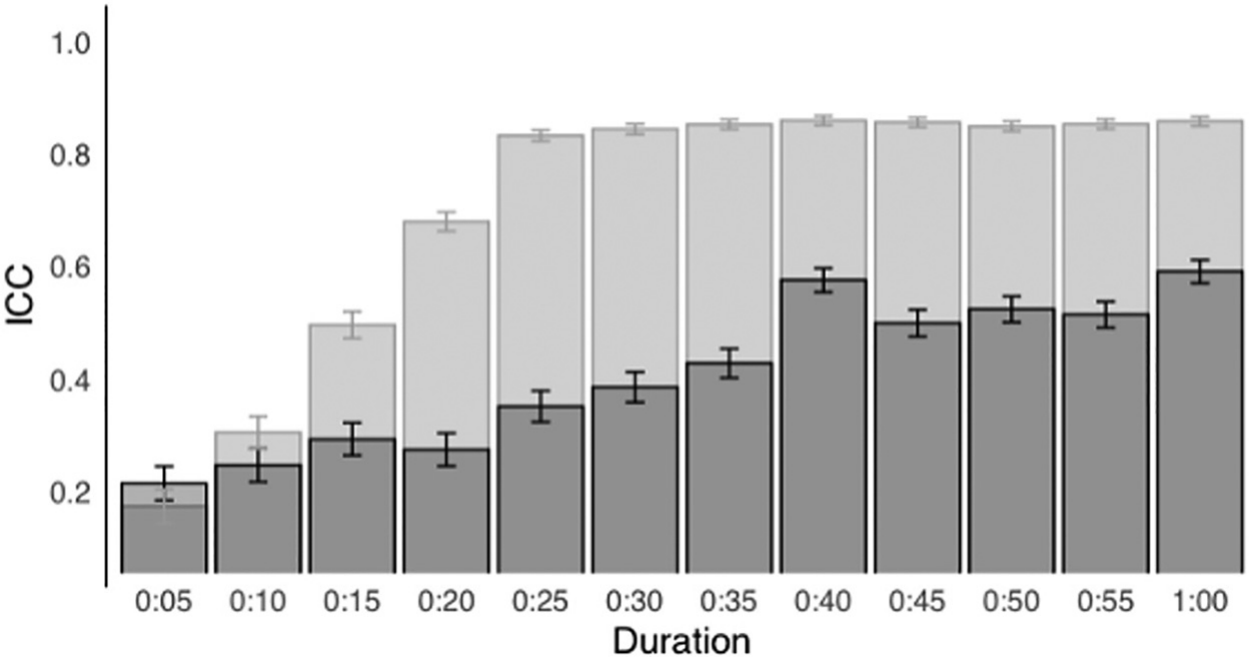
Intraclass Coefficient for Oxygen Saturation Measurements Versus Duration of Measurements^a^ Abbreviation: ICC, intraclass coefficient; SQI, signal quality index. ^a^ N=3,823. Dark bars represent intraclass correlation coefficient when including all seconds regardless of their SQI value (Q1). Light bars represent intraclass correlation coefficient when only seconds with SQI≥90% were included (Q2). Less than 1% of the records were excluded in the first 15 seconds because those records did not have enough seconds matching the quality criteria. The bars indicate the intraclass correlation coefficient values over varying spot measurement durations.

The ICC (Q1) for SpO2 measurements for the 60-second duration was 0.58 (95% CI=0.56, 0.60) (Figure 3). The ICC (Q1) for the median SpO2 for only the first 5 seconds was 0.2 (95% CI=0.17, 0.23). In general, the ICC (Q1) value increased with increasing duration of up to 40 seconds. After 40 seconds, the ICC (Q1) plateaued at 0.5. The ICC (Q2) was much higher than the ICC (Q1) for all durations except in the first 10 seconds when the repeatability was very low for both. The ICC (Q2) plateaued after 35 seconds at 0.85. For the full 60-second duration, the ICC (Q3) was even higher at 0.91 (95% CI=0.90, 0.91); (n=3,230).

The median of SpO2 in the study was 97.7% for both recordings (IQR: 96.2%–98.9% for SpO2-1 and IQR: 95.9%–98.8% for SpO2-2). For only the first 35 seconds, the median was 98.0% (IQR: 96.5%–99.1% for the best recording and IQR: 96.3%–99.1% for the second best).

## DISCUSSION

In this article, we report the repeatability of pulse oximetry measurement in children during triage at 2 hospitals in Uganda based on 2 60-second spot measurements of SpO2 per child. The repeatability value for the full minute (ICC=0.85) was representative of high repeatability; however, shorter periods of observation, lower signal quality, and lower oxygen saturation reduced repeatability. When low-quality data were included, there was a large reduction in repeatability and measurement agreement. Repeatability increased as the duration of recordings lengthened until the ICC plateaued at 35 seconds. Improved repeatability of SpO2 recordings can be achieved with practices, such as optimizing signal quality, extending the duration of the recording to at least 35 seconds, and with repeat observations when SpO2 measurements are below 90%.

### Implications for Clinical Care

The expansion of digital health tools in LMICs has been accelerated by the COVID-19 pandemic.^20^^–^^22^ Pulse oximeters are in demand in low-resource settings, and it is critical that they are used appropriately.^8^^,^^23^ The integration of pulse oximetry into the digital health landscape is an opportunity to design intelligent systems that include averaging for spot measurements, filtering based on quality criteria, and enforcing minimum measurement durations. Understanding the factors that reduce repeatability will facilitate improved device design and optimize clinical procedures and training.

We have shown that both SQI and duration of measurement should be optimized to improve repeatability. We observed that without quality filtering, 2 SpO2 measurements done by the same observer using the same device on the same patient over a short period of time have only moderate repeatability based on an ICC of 0.5. There are multiple factors that will reduce the repeatability of an SpO2 measurement in addition to the accuracy of the device itself. These factors include the short-term variation within the patient (within-subject variation), the variations in how observers perform the measurement (within- and between-observer variation), and other variations in filtering, averaging, or rounding of measurements.^24^

Both SQI and duration of measurement should be optimized to improve repeatability of SpO2 measurement.

It may take time for the measurement to stabilize when applying a pulse oximeter to a child due to the delay in signal processing algorithms in the device.^25^ This stabilization period will vary between devices, subjects (based on their oxygenation status), and operators. Poor application of the sensor (sensor exposure to external light), a restless child (sensor motion), a child with poor circulation or cold limbs (low perfusion), or a child that is not clinically stable (changing SpO2 or HR), are all recognized by the Masimo pulse oximeter and contributed to a lower SQI (Supplement) and thus delays in stabilization of the measurement. However, we have shown that choosing an observation obtained over a short time frame (initial 5 seconds) will lead to poor repeatability and greater variability than a longer measurement, even when data collectors are trained to wait for this stabilization and design affordance features are used (i.e., a green-colored background indicating high-quality data, SQI≥90%). The increased variability will most likely lead to reduced accuracy in the measurement, which can ultimately affect prompt identification and treatment of hypoxemia.

### Limitations

This study was performed at only 2 institutions and included only 14 observers. All pulse oximeter data were collected with identical pulse oximeter models and a custom application that was designed to improve observer performance. There may have been some dependency between observers, but initial analysis of the ICC (Q1) using a multiple-rater model showed no significant difference, so this dependency was not included in the final analysis. Due to the range of ages involved in this study, the body site of the pulse oximetry measurement varied. The site was chosen by the nurses using their clinical training to achieve a high-quality signal. This site would have been unlikely to change between the 2 measurements, but it was not recorded, potentially posing another challenge to repeatability.^26^ Further, external validity of the study may be limited as the study sites in Uganda are at an altitude of 1100–1200 m above sea level. The relative hypoxia at this altitude may have reduced the repeatability due to the nonlinear shape of the oxygen saturation curve. Also, the range of SpO2 values would be shifted lower with a broader range of true saturation values at higher altitudes, which is another contributing factor to reduced repeatability. The repeatability may have been influenced by the reduced accuracy of pulse oximetry in individuals with dark skin pigmentation.^27^ The range of SpO2 values measured may have biased ICC values compared to observations at sea level. The largest differences in SpO2 were seen at low SpO2 values, but this is to be expected given that the majority of the SpO2 values were high (>90%) and on the flat part of the oxygen dissociation.^28^ Additionally, this secondary analysis did not consider clinical outcomes nor attempt to define an acceptable ICC value for adequate repeatability. ICC values have previously been defined as the following: less than 0.2—slight repeatability, between 0.2 and 0.4—low repeatability, between 0.4 and 0.7—moderate repeatability, between 0.7 and 0.9—high repeatability, and greater than 0.9—very high repeatability.^24^ Using this scale, this study showed that repeatability increased from low to moderate when using 35 seconds of data versus a shorter time frame. A further increase in high repeatability occurred when only high-quality data were used to calculate the median reported SpO2. This secondary analysis was conducted using the Masimo iSpO2 device, which provides good accuracy within the International Organization for Standardization standards. Other models of pulse oximeters may have different results, especially if they do not provide adequate feedback to the user about the current quality of the measurement.^24^ In addition, the iSpO2 may not be the ideal device for many low-resource settings as it requires a mobile device and costs US$330. Lastly, poor repeatability of pulse oximetry measurements may not necessarily indicate poor accuracy, and good repeatability, on the other hand, does not necessarily equal good accuracy; one should not be taken as a substitute for the other as there are many forms of uncertainty, including user’s technique and fluctuations in vital signs between measurements.^24^

## CONCLUSION

This study demonstrates that shorter periods of observation, lower signal quality, and lower oxygen saturation levels reduce repeatability of pulse oximetry measurements. Repeatability of observations is critical for making optimal clinical decisions but also essential when performing device validation. These results should inform training for health workers who perform pulse oximetry. For example, observers (or a pulse oximetry device) should average SpO2 readings for at least 35 seconds of high-quality data to obtain a repeatable measurement. Future studies should concentrate on within-observer uncertainty in different settings and with different devices and evaluate other causes of uncertainty such as within-subject variability.

## Supplementary Material

GHSP-D-22-00544-supplement.pdf

## Notes

**Peer Reviewed**

**First published online:** August 17, 2023.
