# Supplementary material for: Repeatability of Pulse Oximetry Measurements in Children During Triage in 2 Ugandan Hospitals
Source: Glob Health Sci Pract. 2023 Aug 28;11(4):e2200544. doi: 10.9745/GHSP-D-22-00544 (PMC10461707; doi:10.9745/GHSP-D-22-00544)

Supplement to: Asdo A, Mawji A, Agaba C, et al. Repeatability of pulse oximetry measurements in children during triage in 2 Ugandan hospitals. *Glob Health Sci Pract*. 2023;11(4):e2200544. https://doi.org/10.9745/GHSP-D-22-00544

SUPPLEMENT

The Signal Quality Index (SQI) calculated during the pulse oximetry data collection for the Smart Triage study has a range of 0%–100%, with 100% considered to be a perfect quality signal. The algorithm takes the following input at 1Hz: HR (heart rate), SpO_2 (oxygen saturation), PFI (perfusion index) and Masimo flags for no sensor detected, defective sensor, sensor off patient, search for pulse, low Signal IQ, and low perfusion.

The SQI algorithm works by starting with 100% and subtracting penalty values from this based on any problems found. The SQI is never set below 0%.

Pseudo code for the algorithm (run at 1Hz):

1. If there is no HR calculated (HR = 0), SQI = 0, skip all below steps.
2. Set SQI = 100.
3. Check the Masimo flags:
    a. If no sensor detected, defective sensor, or sensor off patient, SQI = 0.
    b. If artifacts flag within the last 1.2 seconds, SQI = SQI – 40%.
    c. If searching for pulse, SQI = SQI – 60%.
    d. If low perfusion flag, SQI = SQI – 50%.
4. Calculate PFI as a percentage out of 20.
    a. If PFI% ≤ 2.5% then SQI = 0.
    b. Otherwise, if PFI% ≤ 6% then SQI = SQI – 40%.
    c. Otherwise, if PFI% < 25% then give a small penalty according to the following:
    Calculate P as the percent of the way from 25% down to 6% using the formula:

$$P = \frac{25 - PFI}{25 - 6}$$

    Then multiply this value by 10 to get PP (0 < PP < 10), then SQI = SQI – PP.
5. Check SpO_2 variability. The last 30 seconds of SpO_2 data is stored to look at variability.
    a. Calculate the interquartile range of SpO_2, SIQR.
    b. Calculate the median SpO_2, SM.
    c. If $\frac{SIQR}{2 * SM} > 10.0$, then SQI = SQI – VS.
6. Check HR variability. The last 30 seconds of HR data is stored to look at variability.
    a. Calculate the interquartile range of HR, HIQR.
    b. Calculate the median HR, HM.
    c. If $\frac{HIQR}{2 * HM} > 10.0$, then SQI = SQI – VH.

The resulting SQI from the above algorithm is displayed as a colour in the data collection app. Below is a diagram of the colours used. Only green seconds of data (SQI ≥ 90) are used in calculating an overall median HR and SpO_2 for the spot-check measurement.

Supplement to: Asdo A, Mawji A, Agaba C, et al. Repeatability of pulse oximetry measurements in children during triage in 2 Ugandan hospitals. *Glob Health Sci Pract*. 2023;11(4):e2200544. https://doi.org/10.9745/GHSP-D-22-00544

Figure: SQI color coding feedback in the data collection app

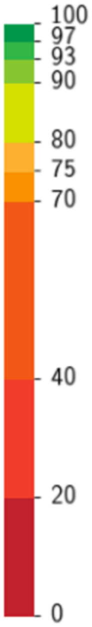

Supplement: GHSP-D-22-00544-supplement.pdf [file GHSP-D-22-00544-supplement.pdf]
